# Functional insights of nucleocytoplasmic transport in plants

**DOI:** 10.3389/fpls.2014.00118

**Published:** 2014-04-02

**Authors:** Kentaro Tamura, Ikuko Hara-Nishimura

**Affiliations:** Department of Botany, Graduate School of Science, Kyoto UniversityKyoto, Japan

**Keywords:** nucleocytoplasmic transport, nuclear transport receptors, nucleoporins, nuclear pore complex, *Arabidopsis thaliana*

## Abstract

Plant nucleocytoplasmic transport beyond the nuclear envelope is important not only for basic cellular functions but also for growth, development, hormonal signaling, and responses to environmental stimuli. Key components of this transport system include nuclear transport receptors and nucleoporins. The functional and physical interactions between receptors and the nuclear pore in the nuclear membrane are indispensable for nucleocytoplasmic transport. Recently, several groups have reported various plant mutants that are deficient in factors involved in nucleocytoplasmic transport. Here, we summarize the current state of knowledge about nucleocytoplasmic transport in plants, and we review the plant-specific regulation and roles of this process in plants.

## INTRODUCTION

The nucleus is the most prominent organelle in eukaryotes and is surrounded by the nuclear envelope, which provides a controlled barrier between the nucleoplasm and the cytoplasm. The nuclear envelope consists of a double membrane spanned by nuclear pore complexes (NPCs), which form channels, allowing the passive diffusion of small molecules. Macromolecules larger than ~40 kDa are transported actively across the nuclear envelope through the NPC in a regulated manner (Grossman et al., 2012; Raices and D’Angelo, 2012). How the rapid, bidirectional trafficking of thousands of specific cargoes through the NPC is achieved has been the subject of intense study (Mosammaparast and Pemberton, 2004). In addition to bulk transport of constitutive nuclear proteins, changes in gene expression generally require the controlled import/export of key signaling molecules to/from the nucleus (Stewart, 2007; Merkle, 2011), indicating that nucleocytoplasmic transport is a highly dynamic process.

The first step in nucleocytoplasmic transport is the recognition of cargo molecules by specific transport receptors, including importins and exportins (Bednenko et al., 2003; Mosammaparast and Pemberton, 2004; Stewart, 2007). Following formation of the cargo–transport receptor complex, the NPC mediates translocation of this complex through the nuclear envelope. Such selective transport of molecules across the nuclear envelope plays an important role not only in basic cellular activity, but also in cellular differentiation and responses to environmental signals (Raices and D’Angelo, 2012; Parry, 2013). Recent investigations of nucleocytoplasmic transport have revealed unique roles and unexpected layers of regulation of this process in plants (Meier and Somers, 2011). In this review, we summarize studies involving mutants defective in nucleocytoplasmic transport factors, and we discuss the key roles of the nucleocytoplasmic transport system in plant cells.

## IMPORTIN β FAMILY IN *Arabidopsis thaliana*

Importin β (or karyopherin β) family proteins are major nuclear transport receptors that interact with Ran small GTPase and mediate the nuclear transport of specific cargoes (Lott and Cingolani, 2011; Merkle, 2011). These proteins are also referred to as importins or exportins depending on whether they mediate cargo import into, or export out of, the nucleus. The structures of importin β proteins are characterized by a similar series of helical HEAT repeats, which are approximately 40 residues in length (Strom and Weis, 2001; Xu et al., 2010; Lott and Cingolani, 2011). The fundamental repeat unit is a right-handed superhelical structure consisting of a hairpin comprising two β helices. Each hairpin is connected to the next by a linker region. Crystal structural analysis has indicated that mammalian importin β is composed of 19 HEAT repeats and uses extensive interaction interfaces to associate with different cargoes (Cingolani et al., 1999, 2002; Lee et al., 2003).

**FIGURE 1:**
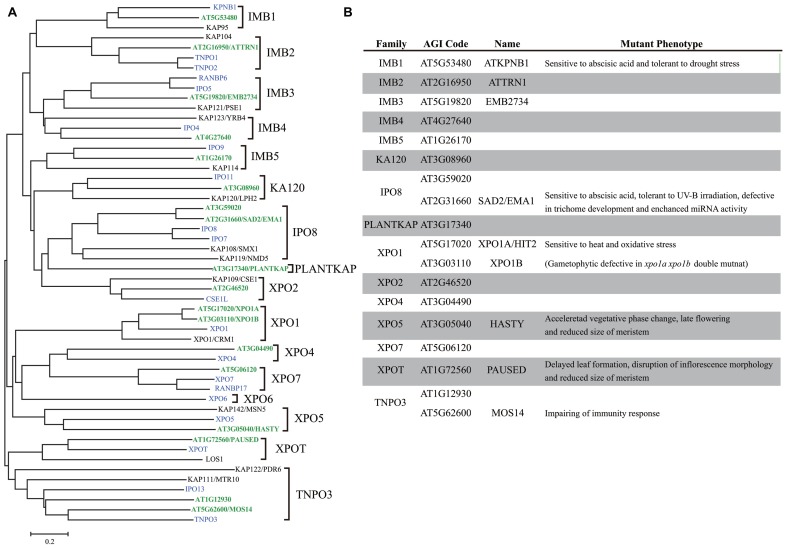
**Comparison of importin β family proteins in *Arabidopsis*, human, and yeast. (A)** Phylogenetic tree of importin β family in *Arabidopsis* (green), human (blue), and yeast (black) constructed using the neighbor joining method. **(B)** *Arabidopsis* importin β family proteins.

The *Arabidopsis thaliana* genome encodes 18 importin β proteins (**Figure 1**), while the *Saccharomyces cerevisiae* genome encodes 14 importin β proteins and the *Homo sapiens* genome encodes more than 20 of these proteins (Goldfarb et al., 2004). At least 16 subfamilies of importin β, each containing representatives from eukaryotic subgroups, have been identified (**Figure 1**), suggesting that these importin β subfamilies were established prior to eukaryotic radiation (O’Reilly et al., 2011). Plant genomes lack the *exportin 6* (*XPO6*) gene family, while embryophyte-specific sequences have been identified, which were designated PLANTKAP (At3g17340 in *A. thaliana*; O’Reilly et al., 2011; **Figure 1**).

## IMPORTIN α FAMILY IN *A. thaliana*

The best understood function of importin α is to serve as an adaptor that links classical nuclear localization signal (cNLS)-containing proteins to importin β, which interacts with the ternary complex at the NPC. Importin α comprises two functionally and structurally distinct domains, namely, the flexible N-terminal importin β-binding (IBB) domain and a C-terminal domain that consists of eight to nine tandem armadillo (ARM) repeats (Goldfarb et al., 2004; Marfori et al., 2011). The stacking of helical ARM repeats generates a right-handed superhelical structure, forming two separate cNLS-binding sites (the major and minor sites; Conti et al., 1998; Marfori et al., 2011). Chang et al. (2012) determined the crystal structure of rice importin α1a at 2-Å resolution. Consistent with *S. cerevisiae* and *H. sapiens* proteins, *Oryza sativa* importin α preferentially binds to the prototypical cNLS from SV40 large T-antigen at the major nuclear localization signal (NLS) binding site. On the contrary, two plant-specific NLSs (Kosugi et al., 2009) bind to the minor site of rice importin α. Interestingly, mouse importin α binds to these plant-specific NLSs at the major site, suggesting that plant importin α has plant-specific features to mediate nuclear import.

**FIGURE 2:**
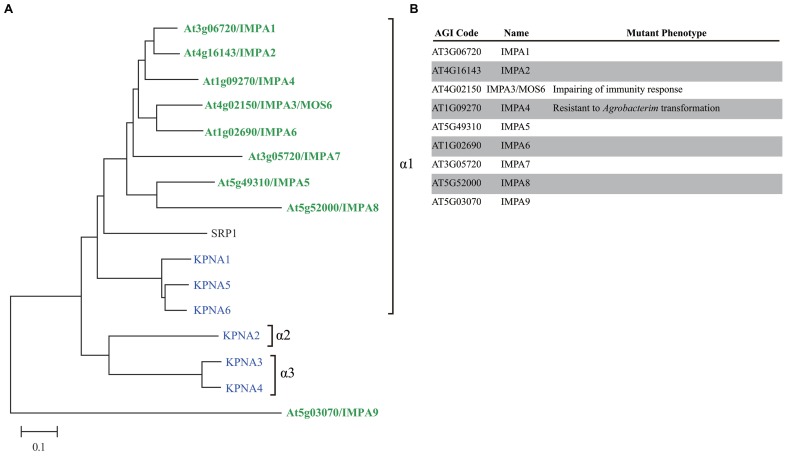
**Comparison of importin α family proteins in *Arabidopsis*, human, and yeast. (A)** Phylogenetic tree of importin α family in *Arabidopsis* (green), human (blue), and yeast (black) constructed using the neighbor joining method. **(B)** *Arabidopsis* importin α family proteins.

The number of importin α family genes has increased over the course of evolution; yeast has a single importin α gene, *Drosophila melanogaster* has four genes, and *H. sapiens* has six genes (Mason et al., 2009; Yasuhara et al., 2009; **Figure 2**). *H. sapiens*, *D. melanogaster*, and most animal importin α genes fall into one of three conserved clades, i.e., α1, α2, and α3 (Mason et al., 2002, 2009). By contrast, plant and fungal importin α belong to α1 and a non-conventional family (**Figure 2**), suggesting that the importin α1 gene may be the earliest progenitor of importin α, and α2 and α3 are metazoan specific importin α. *A. thaliana* has eight importin α proteins (IMPA1–7 and 9) that contain both IBB domains and ARM repeats and a single protein (IMPA8) that contains only ARM repeats. Based on sequence similarity, *A. thaliana* IMPA9 is likely to be a non-conventional importin α that does not fall into the α1, α2, or α3 groups (**Figure 2**). *A. thaliana* IMPA1 binds to all three types of NLS *in vitro*, which were identified in maize and SV40 (Smith et al., 1997). Moreover, IMPA1–4 interact with *A. thaliana* CAS (cellular apoptosis susceptibility), which is required for recycling importin α from the nucleus to the cytosol, indicating that nucleocytoplasmic transport is functionally conserved in plants (Haasen and Merkle, 2002).

## STRUCTURE OF THE NUCLEAR PORE COMPLEX

The NPC is a macromolecular assembly of protein subcomplexes that serves as a key regulator of molecular trafficking between the cytoplasm and the nucleus (Grossman et al., 2012; Parry, 2013; Tamura and Hara-Nishimura, 2013). Extensive studies have identified more than 30 different nucleoporin proteins that serve as building blocks of the NPC (Rout et al., 2000; Cronshaw et al., 2002; Tamura et al., 2010). By contrast to other nuclear envelope proteins, NPC components are evolutionarily conserved between yeast, animals, and plants, indicating that these NPCs share a common progenitor (Devos et al., 2006). Based on their function in the NPC, nucleoporins are classified into two groups, including FG (phenylalanine–glycine) repeat-containing nucleoporins and scaffold nucleoporins. The FG-repeat domain is composed of multiple FG-rich peptides and is natively unfolded (Denning et al., 2003). FG nucleoporins account for as much as one-third of the molecular mass of the NPC. Since nuclear transport receptor–cargo complexes dock to the NPC by binding to their FG domains, FG nucleoporins are key determinants of nucleocytoplasmic transport. Scaffold nucleoporins form biochemically stable NPC subcomplexes that appear to play important roles as building blocks during NPC biogenesis on the nuclear membrane. NPC core scaffold proteins consist of cage-like structures, which is similar to the structure of coated vesicles (Bonifacino and Glick, 2004; Strambio-De-Castillia et al., 2010). This characteristic similarity suggests that a simple membrane-curving module is the ancient common ancestor of NPCs and coated vesicles (Devos et al., 2006; Alber et al., 2007). In addition to their roles in nucleocytoplasmic transport, NPCs and individual nucleoporins are involved in a large number of cellular processes, including kinetochore and spindle assembly, regulation of gene expression, chromatin organization, and DNA repair (Strambio-De-Castillia et al., 2010; Bukata et al., 2013; Van de Vosse et al., 2013; **Table 1**).

**Table 1:** Identified plant nucleoporin mutants.

Nup name	AGI code	Other name	Related pathway	Reference
Nup160	At1g33410	SAR1	Flowering, mRNA export, immunity, cold stress, and auxin signaling	Dong et al. (2006a), Parry et al. (2006), Robles et al. (2012), Wiermer et al. (2012)
Nup96	At1g80680	SAR3/MOS3	Flowering, mRNA export, immunity, and auxin signaling	Zhang and Li (2005), Parry et al. (2006)
TPR/NUA	At1g79280		Flowering, mRNA export, miRNA metabolism, and auxin signaling	Jacob et al. (2007), Xu et al. (2007)
Nup58		TCU1	Flowering	Ferrandez-Ayela et al. (2013)
Nup62			Flowering	Zhao and Meier (2011)
Nup136/Nup1			Flowering and mRNA export	Lu et al. (2010), Tamura et al. (2010)
ELYS		HOS1	Flowering, mRNA export, and cold stress	Dong et al. (2006b), Lazaro et al. (2012), Jung et al. (2013), MacGregor et al. (2013)
Nup133			Symbiosis	Kanamori et al. (2006), Binder and Parniske (2014)
Nup75/85			Symbiosis	Saito et al. (2007), Binder and Parniske (2014)
Seh1		NENA	Symbiosis, mRNA export and immunity	Groth et al. (2010), Wiermer et al. (2012)
Nup88		MOS7	Immunity	Cheng et al. (2009)
Nup214		LNO1	Embryo development	Braud et al. (2012)
GLE1			Embryo development	Braud et al. (2012)

## DEVELOPMENT

### THE ROLE OF EXPORTINS IN PLANT DEVELOPMENT

The first reported *A. thaliana* importin β family mutant was *hasty* (*hst*; Telfer and Poethig, 1998). In a forward genetic screen, *hst* was originally identified as a mutant defective in the transition between developmental phases. The *hst* mutant exhibits pleiotropic phenotypes, including reduced size of the shoot apical meristem, accelerated vegetative phase change, late flowering under short day conditions, and reduced fertility (Bollman et al., 2003), suggesting that HST is essential for various developmental pathways in plants. The *HST* gene is the ortholog of *H. sapiens exportin 5* (*XPO5*) and the *S. cerevisiae* bidirectional transporter *MSN5* (Bollman et al., 2003). In addition, HST interacts with Ran in yeast two-hybrid assays and localizes to the nuclear periphery (Bollman et al., 2003). Another exportin mutant, *paused* (*psd*), was isolated by two different groups (Telfer et al., 1997; Hunter et al., 2003; Li and Chen, 2003). The *psd* mutant exhibits transiently disrupted organization of the shoot apical meristem, but the timing of the transition to the adult phase of vegetative development is not significantly altered in this mutant (Telfer et al., 1997). *PSD* encodes an ortholog of exportin-t (XPOT), which mediates nuclear export of tRNAs in yeast (Hellmuth et al., 1998) and human (Kutay et al., 1998).

The different phenotypes observed between the *hst* and *psd* exportin mutants can be accounted for by their impaired nuclear export of different cargoes. The *hst* mutation leads to reduced accumulation of most micro RNAs (miRNAs) but has no effect on the accumulation of tRNAs or endogenous small interfering RNAs (Park et al., 2005). On the contrary, the *psd* mutation results in compromised tRNA-Tyr processing but does not affect the accumulation or nuclear export of miRNAs (Park et al., 2005). These results clearly indicate that HST and PSD do not share RNA cargoes for nuclear export. Therefore, multiple nuclear export pathways for these small RNAs are required for plant development.

*Arabidopsis thaliana* has two loci for exportin 1 (*XPO1* in vertebrates), designated as *XPO1A* (At5g17020) and *XPO1B* (At3g03110); the corresponding proteins share 86% identity (Blanvillain et al., 2008). Single mutants for *XPO1A* and *XPO1B* appear normal, indicating that these genes function redundantly. However, a double mutant homozygote has not been recovered (Blanvillain et al., 2008). Consistent with this observation, cotransmission of mutant alleles of these genes is abolished through the female and is strongly reduced through the male. Female gametophytes of the double mutant show defects ranging from early developmental arrest to disorganized cellular constitution. It was therefore concluded that a maternal copy of *XPO1* is required to establish a viable embryo (Blanvillain et al., 2008).

### FLOWERING

As expected, since nucleoporin is involved in fundamental cellular functions, many nucleoporin mutants exhibit pleiotropic phenotypes at both the reproductive and vegetative developmental stages. An early flowering phenotype is commonly observed in various nucleoporin mutants, including *nup58* (Ferrandez-Ayela et al., 2013), *nup62* (Zhao and Meier, 2011), *nup96/sar3/mos3* (Zhang and Li, 2005; Parry et al., 2006), *nup136/nup1* (Lu et al., 2010; Tamura et al., 2010), *nup160/sar1* (Dong et al., 2006b; Parry et al., 2006), *hos1/elys* (Ishitani et al., 1998; Lazaro et al., 2012; Jung et al., 2013; MacGregor et al., 2013), and *tpr/nua* (Jacob et al., 2007; Xu et al., 2007). Xu et al. (2007) found that the *tpr/nua* mutation affects the expression of flowering-related genes. The expression of floral repressor genes (*FLC* and *MAF4*) is reduced in the *tpr/nua* mutant, while the expression of floral activator genes (*FT*, *SOC*, *LFY*, *MYB33*, and *MYB65*) is increased, compared to wild type. Jacob et al. (2007) identified TPR/NUA as a suppressor of *FLC* expression. The authors also found that *tpr/nua* flowers earlier than the *flc* null mutant, suggesting that FLC-dependent and -independent flowering pathways are regulated by TPR/NUA.

The function of HOS1/ELYS in flowering pathway has been well studied. Genetic analysis with *hos1 flc* double mutant suggested that the HOS1/ELYS regulates flowering time independent of FLC (Lazaro et al., 2012; Lee et al., 2012a). Moreover, it was found that HOS1/ELYS and ubiquitinated-CONSTANS (CO) physically interact, and HOS1/ELYS regulates CO abundance, particularly during the daylight period (Lazaro et al., 2012). These results suggest that HOS1/ELYS plays a key role in the proteasome-dependent degradation of CO at nuclear pore for control of flowering time (Lazaro et al., 2012; Joon Seo et al., 2013). MacGregor et al. (2013) reported that *hos1/elys* is affected in circadian clock function, exhibiting a long-period phenotype. Interestingly, Lee et al. (2012b) demonstrated that alternative HOS1 splicing variant is crucial for regulating flowering time, and expresses in a photoperiod-dependent manner.

On the contrary, it was also reported that HOS1/ELYS directly regulates FLC transcription at the chromatin level through interactions with FVE, a histone binding protein (Lee et al., 2012a), and histone deacetylase 6 (HDA6; Jung et al., 2013). FVE and HDA6 interact with each other to function in chromatin silencing in *A. thaliana* (Gu et al., 2011). HOS1/ELYS also interacts with HDA6 and inhibits the binding of HDA6 to *FLC* chromatin. Moreover, cold treatment induces *FLC* expression by activating HOS1, which inhibits the association of HDA6 with *FLC* chromatin, resulting in delayed flowering. Therefore, HOS1/ELYS is thought to act as a chromatin-remodeling factor for *FLC* regulation in response to cold stress. Similar NPC-dependent gene regulation systems that function via chromatin remodeling have been reported in other organisms. In mammalian cells, the nuclear basket component in NPC plays a fundamental role in chromatin organization (Krull et al., 2010). Loss of a nucleoporin induces chromatin condensation all along the nucleoplasmic side of the nuclear envelope, suggesting that NPC maintains pore-associated chromatin in an open state (Grossman et al., 2012). *S. cerevisiae* Nup170 binds to specific chromatin domains to promote transcriptional repression through its interactions with chromatin-remodeling complex (Van de Vosse et al., 2013). Interactions between chromatin and nucleoporins may represent an initial event in the regulation of gene expression, because this process occurs independently of any preceding transcription (Schmid et al., 2006). Taken together, these results suggest that HOS1/ELYS in NPC also provides a platform for transcriptional regulation to control flowering time in plants.

### ESSENTIAL NUCLEOPORINS

Given their fundamental functions in cellular activities, nucleoporins are thought to be essential for plant viability. However, only a few essential nucleoporins have been identified in *A. thaliana*. A certain allele of *nup136/nup1* mutant exhibits gametophytic or embryonic lethal (Lu et al., 2010). A partial loss of *Nup88*/*MOS7* produces variable phenotypes, while a complete loss of *Nup88/MOS7* causes lethality (Cheng et al., 2009). This result is consistent with the lethal phenotype of a null *nup88* mutant in *D. melanogaster* (Uv et al., 2000). Nup214, which provides binding sites for mRNA export factors, was also found to be essential. A mutation in *Nup214* abolishes the first asymmetrical cell division during early embryogenesis, resulting in an arrest in embryo development (Braud et al., 2012). Furthermore, a mutation in GLE1, which is thought to work in conjunction with Nup214 in the mRNA export pathway, also leads to embryonic lethality (Braud et al., 2012). In *S. cerevisiae*, large-scale deletion analysis has already identified many nucleoporins that are essential for cellular viability (Giaever et al., 2002). Further systematic analysis of all *A. thaliana* nucleoporins using T-DNA knockout lines will provide insights into the functional diversity and redundancy of each plant nucleoporin.

## HORMONE AND STRESS SIGNALINGS

### THE ROLE OF IMPORTINS AND EXPORTINS IN HORMONE AND STRESS SIGNALING PATHWAYS

SAD2 [super sensitive to abscisic acid (ABA) and drought2], which is an ortholog of vertebrate importin 7 and 8, was suggested to regulate various hormone and environmental response pathways in *A. thaliana*. The *sad2* mutant exhibits ABA- and stress-hypersensitive induction of luciferase reporter activity (Verslues et al., 2006). Interestingly, *sad2* exhibits ABA hypersensitivity during seed germination and seedling growth, suggesting that SAD2 is involved in nuclear transport of a negative regulator of ABA sensitivity (Verslues et al., 2006). *A. thaliana* contains one gene (At3g59020) homologous to *SAD2* that is in the same clade as importin 7/8 (**Figure 1**). However, knock out of this gene does not duplicate the *sad2* phenotype in response to ABA during seedling growth (Verslues et al., 2006), indicating that SAD2 transports specific cargoes during ABA signaling. In addition to ABA responses, jasmonic acid (JA)-inducible trichome formation is also impaired in the *sad2* mutant (Yoshida et al., 2009). The subnuclear localization of the bHLH transcription factor GLABRA3 (GL3), which promotes trichome formation in response to JA, is disrupted in *sad2*. This suggests that SAD2 regulates (either directly or indirectly) the subnuclear localization of GL3 in response to JA.

The R2R3-type transcription repressor MYB4 is a cargo protein of SAD2 that undergoes nuclear transport (Zhao et al., 2007). MYB4 was previously found to control negative expression of cinnamate 4-hydroxylase (Jin et al., 2000) and, therefore, regulate the synthesis of sinapate esters, which are major photoprotective pigments (Li et al., 1993; Jin et al., 2000). Zhao et al. (2007) demonstrated that *sad2* significantly accumulates UV-absorbing pigments and is more tolerant to UV-B irradiation than the wild type. The authors also found that MYB4 coimmunoprecipitates with SAD2 and its nuclear localization requires functional SAD2, suggesting that SAD2 transports MYB4 to the nucleus to help regulate the plant response to UV-B radiation.

*Arabidopsis thaliana* KPNB1 (At5g53480), which belongs to the IMB1 family, also modulates ABA signaling (Luo et al., 2013). Like the *sad2* mutant, the *kpnb1* mutant exhibits increased ABA hypersensitivity during seed germination and cotyledon development. Moreover, the *kpnb1* mutation increases stomatal closure in response to ABA, reduces the rate of water loss, and substantially increases drought tolerance. However, KPNB1 was proposed to regulate an ABA pathway independently of SAD2. Loss of function of SAD2 results in early flowering and increased expression of ABA-responsive genes (*RD20A* and *RAB18*) under normal conditions (Verslues et al., 2006). On the contrary, the *kpnb1* mutant exhibits late flowering and normal expression of these genes (Luo et al., 2013). These results suggest that KPNB1 transports different cargoes from those of SAD2 to regulate ABA signaling.

As described above, two *A. thaliana XPO1* genes (*XPO1A*, At5g17020 and *XPO1B*, At3g03110) were previously considered to be functionally redundant (Haasen et al., 1999; Merkle, 2003; Blanvillain et al., 2008). However, the *hit2* (*heat intolerant 2*) mutant, in which a single *XPO1A* gene is mutated, shows a defect in basal but not acquired thermotolerance (Wu et al., 2010). The *hit2* mutant is also sensitive to methyl viologen-induced oxidative stress, and the survival of *hit2* seedlings in response to heat stress is affected by light conditions. It was therefore concluded that the *hit2* phenotype is attributable to the lack of a sufficient response to heat-induced oxidative injury (Wu et al., 2010). This study clearly indicates that XPO1A has its own specific function and cargoes, which are not necessary for normal growth but are important for plant survival under conditions of sustained or sudden heat stress.

### COLD SIGNALING

There is significant evidence to demonstrate that nucleoporins are required for cold responses. In a screen for altered expression of cold-induced reporter genes, Zhu and colleagues identified several mutants, including *nup160/sar1* (Dong et al., 2006b) and *hos1/elys* (Ishitani et al., 1998; Lee et al., 2001; Dong et al., 2006a). The *nup160/sar1* mutant impairs cold-responsive gene expression and is sensitive to chilling stress and defective in acquired freezing tolerance (Dong et al., 2006b). On the contrary, *hos1/elys* exhibits enhanced expression of cold-induced genes in response to low temperature treatment, suggesting that HOS1/ELYS negatively regulates the cold acclimation responses (Lee et al., 2001; MacGregor et al., 2013). Gong et al. (2005) isolated a *cryophyte* mutant, which exhibits an enhanced cold stress-induction of the master regulator of cold tolerance (*CBF2*), and its downstream target genes. The mutant is more tolerant to chilling and freezing stresses but is more sensitive to heat stress. The mutation is found in a DEAD box RNA helicase gene that is identical to the previously identified *low expression of osmotically responsive genes 4* (*LOS4*) locus. The *A. thaliana* LOS4 is the ortholog of *S. cerevisiae* DBP5, which interacts with Nup159 and controls mRNA export (discussed below; Kohler and Hurt, 2007). It is demonstrated that LOS4–GFP fusion localizes at the nuclear rim (Gong et al., 2005), suggesting that *A. thaliana* LOS4 interacts with nuclear pore as observed in *S. cerevisiae* DBP5.

### AUXIN HORMONE SIGNALING

Genetic screening experiments have revealed that nucleoporin is involved in auxin hormone signaling. The *auxin resistance1* (*axr1*) mutant accumulates Aux/IAA proteins, repressors of auxin-regulated transcription, resulting in a pleiotropic phenotype consistent with an overall reduction in the auxin response (Lincoln et al., 1990). Two mutant *suppressor of axr1* (*sar*) lines have been isolated and characterized (Cernac et al., 1997; Parry et al., 2006). Parry et al. (2006) found that *SAR1* and *SAR3* encode Nup160 and Nup96, respectively, which are nucleoporins in the Nup107–Nup160 subcomplex critical for NPC scaffolding. The authors also found that the *sar1* and *sar3* mutants both exhibit impaired nuclear localization of the transcriptional repressor AXR3/INDOLE ACETIC ACID17, suggesting that SAR1 and SAR3 are required for nuclear transport of Aux/IAA proteins in response to auxin signaling. Robles et al. (2012) reported that the *nup160/sar1* mutant exhibits an enhanced ethylene response. Mutation of *ARF7* or *ARF19* almost fully blocks the ethylene hypersensitive phenotype in *nup160/sar1*, suggesting that auxin signaling is responsible for regulating the magnitude of the ethylene response. Jacob et al. (2007) demonstrated that mutation of TPR/NUA, which forms the nuclear basket of the NPC, also alters the auxin sensitivity of the *axr1* mutant to the same level as that of *nup96/sar3*. Importantly, the levels of several miRNAs, some of which regulate the expression of genes involved in auxin signaling, are significantly reduced in *tpr/nua.* This observation suggests that NPC may link miRNA metabolism to auxin signaling.

## PLANT–PATHOGEN INTERACTION

### THE ROLE OF TRANSPORTIN-SR IN IMMUNITY

Serine–arginine rich (SR) proteins are evolutionarily conserved nuclear proteins that play diverse roles in RNA metabolism, including pre-mRNA splicing, non-sense-mediated mRNA decay, and mRNA translation (Reddy, 2004; Long and Caceres, 2009). Transportin-SR (TRN-SR), which belongs to the TNPO3 subfamily (**Figure 1A**), functions as a nuclear import receptor for SR proteins in *S. cerevisiae* (Pemberton et al., 1997) and *H. sapiens* (Kataoka et al., 1999). Interestingly, knockdown of a *TRN-SR* gene (*TSR-1*) in *Caenorhabditis elegans* results in an early embryonic lethal phenotype, indicating that TRN-SR protein is essential for viability (Longman et al., 2000). *A. thaliana* has two *TRN-SR* orthologs (At1g12930 and At5g62600; **Figure 1**), one of which was identified as *MOS14* (modifier of *snc1-1*, 14), which plays a key role in immune responses (Xu et al., 2011). The *A. thaliana mos14* mutant exhibits impaired nuclear localization of several SR proteins, including mRNA splicing factors (Xu et al., 2011). Consistent with this phenotype, altered splicing patterns of auto-activated Resistance (R) genes (*SNC1* and *RPS4*) were observed in *mos14*. The authors also found that mRNA splicing of *Actin1* and *β-tubulin4* occurs normally in *mos14*, suggesting there is an mRNA specificity in MOS14-dependent splicing. Taken together, these results suggest that MOS14 is required for plant immunity through proper splicing of R genes. It would be interesting to know whether the other *A. thaliana* TRN-SR homolog (At1g12930) has different functions and transports different cargoes from MOS14.

### THE ROLE OF IMPORTIN α IN *Agrobacterium tumefaciens* TRANSFORMATION

To date, two *A. thaliana importin α* mutants have been reported. Palma et al. (2005) identified the IMPA3 (At4g02150)-deficient mutant *mos6* (modifier of* snc1, 6*), which exhibits partially suppressed constitutive-resistance responses in *snc1* mutant. In *mos6*, salicylic acid (SA) accumulation is inhibited in response to virulent pathogens, suggesting that MOS6 functions upstream of SA biosynthesis in the resistance-signaling pathway. Bhattacharjee et al. (2008) found that four importin α isoforms (IMPA1–4) interact with *Agrobacterium tumefaciens* virulence proteins (VirD2 and VirE2), which are required for escorting T-DNA into the host’s nucleus. However, only the *impa4* mutant, but not the other (*impa1–3*) mutants, exhibits resistance to *Agrobacterium tumefaciens* transformation. These results suggest that α1 family proteins functionally diverged to acquire specialized roles involving transport of specific cargoes. In further studies, characterization of multiple *impa* knockout mutants might be required for revealing the functional redundancy of IMPA family in plants.

### THE ROLE OF NUCLEOPORINS IN SYMBIOSIS AND IMMUNITY RESPONSES

Three loss-of-function mutants of Nup133, Nup75/85, and Seh1 have been identified in *Lotus japonicus.* These nucleoporin mutants are impaired in both fungal (arbuscular mycorrhizal fungi) and bacterial (rhizobium bacteria) symbioses in a temperature-dependent manner (Kanamori et al., 2006; Saito et al., 2007; Groth et al., 2010). It was also found that *nup85 nup133* double mutants, but not single mutants, exhibit severe temperature dependent growth and developmental defects (Binder and Parniske, 2014). Although the detailed mechanism of these temperature-dependent phenotypes is unclear, the phenotypes of these mutants are consistent with evidence suggesting temperature-dependent plasticity of nuclear pore plugging in *Xenopus laevis* (Stoffler et al., 2003). Intracellular root infection by either endosymbiont is controlled by the activation of calcium and calmodulin-dependent kinase, a conserved regulatory component of symbiosis signaling. However, these nucleoporin mutants fail to exhibit perinuclear calcium spiking in response to Nod factor, a lipo-chitin oligosaccharide, which leads to differentiation of nodule tissues (Stougaard, 2000). NPC may play a role in calcium spiking by acting as a gate for calcium ions or as a regulator of calcium channels on the nuclear membrane. Although calcium-mediated opening and closing of nuclear baskets has been demonstrated in *X. laevis* (Stoffler et al., 1999), the molecular mechanism underlying the role of nucleoporin-dependent calcium spiking in the symbiotic process in *L. japonicus* is unclear.

The *A. thaliana snc1* (*suppressor of npr1-1, constitutive 1*) mutant, which contains a gain-of-function mutation in the R (resistance) gene, shows constitutive activation of disease-resistance responses against pathogens. In a screen for suppressors of *snc1*, Li and colleagues identified several *mos* (*modifier of snc1*) mutants, including *nup96/sar3/mos3* (Zhang and Li, 2005) and *nup88/mos7* (Cheng et al., 2009). Both *mos* mutants have abolished SA accumulation, pathogenesis-related (PR) gene expression, and basal and R-gene-mediated resistance. Importantly, in *nup88/mos7*, nuclear accumulation of snc1 and the defense signaling components NPR1 (non-expresser of PR genes 1) and EDS1 (enhanced disease susceptibility 1) is significantly reduced, while nuclear retention of other tested proteins is unaffected (Cheng et al., 2009). This result suggests that cargo specificity between nuclear proteins and Nup88 is critical for immune responses. Nup96 is a component of the Nup107–Nup160 subcomplex, which is the evolutionarily conserved and largest subcomplex in the NPC. Wiermer et al. (2012) also examined whether other Nup107–Nup160 subcomplex members are involved in immunity. Among eight putative complex members examined, only plants with defects in Nup96, Nup160, or Seh1 are impaired in basal resistance and exhibit a suppressed auto-immunity phenotype in *snc1* (Wiermer et al., 2012). The *nup160/sar1* and *seh1* mutants also exhibit compromised accumulation of EDS1, an essential regulator of basal resistance, as observed in *nup88/mos7* plants. These results suggest that the functions of these nucleoporins partially overlap, but nucleoporins also play specific regulatory roles in plant immune responses.

## RNA METABOLISM

### REGULATION OF miRNA ACTIVITY BY IMPORTIN

A forward genetic screen for miRNA activity led to the identification of the *ema1* (*enhanced miRNA activity1*) mutant, which is allelic to *sad2* (Wang et al., 2011). Wang et al. (2011) found that the levels of endogenous miRNA targets (*SPL3*, *MYB54*, and *CUC2*) were reduced by 40–50% in *sad2/ema1*, indicating that SAD2/EMA1 is involved in general miRNA production. The authors also demonstrated that the miRNA effector complexes in *ema1* contain higher amounts of miRNAs and elevated mRNA cleavage activity compared to the wild type, indicating that EMA1 modulates miRNA activity by influencing the loading of miRNAs into ARGONAUTE1 (AGO1) complexes. On the contrary, *ema1* has no effect on the accumulation of miRNAs or AGO1 on their cytoplasmic or nuclear distribution. These results suggest that SAD2/EMA1 functions as a negative regulator of the miRNA pathway. Although it is unclear whether SAD2/EMA1 directly or indirectly interacts with AGO1 complexes, SAD2/EMA1 may sequester excessive miRNAs to prevent their loading into the AGO1 complex. Alternatively, nuclear-cytoplasmic distribution of SAD2/EMA1 cargoes may be important for the regulation of miRNA activity.

### REGULATION OF mRNA EXPORT AT NUCLEAR PORE

The mRNA-transport receptor complex physically interacts with the FG-repeats of FG nucleoporins, which allow it to overcome the permeability barrier of the NPC (Kohler and Hurt, 2007). An aberrant mRNA export phenotype is widely observed in nucleoporin mutants, including *nup96/sar3/mos3* (Zhang and Li, 2005; Parry et al., 2006), *nup136/nup1* (Lu et al., 2010; Tamura et al., 2010), *nup160/sar1* (Dong et al., 2006b; Parry et al., 2006), *hos1/elys* (MacGregor et al., 2013), *seh1* (Wiermer et al., 2012), and *tpr/nua* (Jacob et al., 2007; Xu et al., 2007). In addition, deficiencies of NPC-associated factors also result in abnormal mRNA export. Mutation in LOS4, whose yeast ortholog is known to interact with nuclear pore and regulate mRNA export (Kohler and Hurt, 2007), results in abnormal mRNAs accumulation in nucleus (Gong et al., 2005). Lu et al. (2010) reported that TREX-2 complex, which is anchored by Nup136/Nup1, is required for mRNA export in *A. thaliana*. Although several components involved in the mRNA export have been isolated, further study will be required for understanding the molecular mechanisms and physiological roles of mRNA export system in plants.

## CONCLUDING REMARKS

As discussed above, many nucleocytoplasmic transport mutants exhibit pleiotropic phenotypes. MacGregor et al. (2013) demonstrated that *los4* and several nucleoporin mutants including *nup160/sar1*, *nup107*, *nua*, and *hos1/elys* all exhibit long-period circadian phenotypes and alterations to clock gene expression. Since many of the signaling pathways are under control of circadian clock, it is raised possibility that impairing in nucleocytoplasmic transport leads indirect effects on the signaling pathways. Moreover, *tpr/nua* was found to increase largely in the amount of mRNA compared with wild type, leading to broad-scale transcriptome alterations (Jacob et al., 2007). This result also suggests that the nucleocytoplasmic transport has indirect effect on the signaling. Further studies are needed in order to differentiate between direct and indirect role of nucleocytoplasmic transport in plants.

Recent years have seen major progress in our understanding of the molecular mechanisms underlying nucleocytoplasmic transport in plant cells. It is now firmly established that these transport systems are responsible for developmental and signaling pathways that are indispensable for plant growth. Although nucleocytoplasmic transport is an evolutionarily conserved system in eukaryotic cells, plants appear to have developed functionally divergent cargoes and regulatory mechanisms, especially in response to environmental signaling. Achieving a deeper understanding of the mechanisms by which nuclear transport receptors exert these functions will require the identification of the cargo molecules that are transported by these nuclear transport receptors.

## Conflict of Interest Statement

The authors declare that the research was conducted in the absence of any commercial or financial relationships that could be construed as a potential conflict of interest.
